# Ca^2+^/Calmodulin-Dependent AtSR1/CAMTA3 Plays Critical Roles in Balancing Plant Growth and Immunity

**DOI:** 10.3390/ijms19061764

**Published:** 2018-06-14

**Authors:** Peiguo Yuan, Liqun Du, B. W. Poovaiah

**Affiliations:** 1 Laboratory of Molecular Plant Science, Department of Horticulture, Washington State University, Pullman, WA 99164-6414, USA; peiguo.yuan@wsu.edu; 2 College of Life and Environmental Sciences, Hangzhou Normal University, Hangzhou 310036, China

**Keywords:** calcium signaling, plant immune response, plant growth and development, AtSR1/CAMTA3

## Abstract

During plant-pathogen interactions, plants have to relocate their resources including energy to defend invading organisms; as a result, plant growth and development are usually reduced. *Arabidopsis* signal responsive1 (AtSR1) has been documented as a negative regulator of plant immune responses and could serve as a positive regulator of plant growth and development. However, the mechanism by which AtSR1 balances plant growth and immunity is poorly understood. Here, we performed a global gene expression profiling using Affymetrix microarrays to study how AtSR1 regulates defense- and growth-related genes in plants with and without bacterial pathogen infection. Results revealed that AtSR1 negatively regulates most of the immune-related genes involved in molecular pattern-triggered immunity (PTI), effector-triggered immunity (ETI), and in salicylic acid (SA)- and jasmonate (JA)-mediated signaling pathways. AtSR1 may rigidly regulate several steps of the SA-mediated pathway, from the activation of SA synthesis to the perception of SA signal. Furthermore, AtSR1 may also regulate plant growth through its involvement in regulating auxin- and BRs-related pathways. Although microarray data revealed that expression levels of defense-related genes induced by pathogens are higher in wild-type (WT) plants than that in *atsr1* mutant plants, WT plants are more susceptible to the infection of virulent pathogen as compared to *atsr1* mutant plants. These observations indicate that the AtSR1 functions in suppressing the expression of genes induced by pathogen attack and contributes to the rapid establishment of resistance in WT background. Results of electrophoretic mobility shift assay (EMSA) and chromatin immunoprecipitation (ChIP)-PCR assays suggest that AtSR1 acts as transcription factor in balancing plant growth and immunity, through interaction with the “CGCG” containing CG-box in the promotors of its target genes.

## 1. Introduction

Ca^2+^ ions act as an essential second messenger mediating plant growth and development, as well as the establishment of appropriate responses to biotic and abiotic stresses [1,2,3]. *Arabidopsis* Signal Responsive 1 (AtSR1), also known as Calmodulin-binding Transcriptional Activator 3 (CAMTA3), is a Ca^2+^/calmodulin (CaM)-regulated transcription factor (TF), which is involved in decoding Ca^2+^ signals triggered by endogenous or exogenous stimulus, into transcriptional reprogramming to activate proper responses in plants [4,5]. AtSR1 is reported to be a suppressor of plant immune responses through its interaction with the promotor of defense-related genes, such as EDS1 and NDR1, and the repression of their transcription [4,6,7,8,9].

It is well known that resistance to pathogens comes with a price in terms of compromised growth and productivity, and this phenomenon is observed in the *atsr1* mutant plants [10,11]. It seems that AtSR1 represses the plant immune responses to maintain optimal plant growth and development when there is no challenge from pathogens. Several endogenous phytohormones are known to be involved in regulating plant growth. Among them, auxin (AUX) and brassinosteroids (BRs) play key roles in enhancing plant growth and development [12,13]. Indole-3-acetic acid inducible genes products (*IAAs*, also known as Auxin Resistant genes, *AUX*, 29 IAA/AUX homologs exist in *Arabidopsis* genome), together with TOPLESS (TPLs), serve as transcription repressors by interacting with auxin-responsive transcription factors (ARF, 23 homologs in *Arabidopsis*) to keep them inactive [14,15,16,17]. Central to the auxin perception is that auxin, mostly IAA, acts as a molecular glue to join AUX/IAA proteins to TIR1/AFB in the E3 ubiquitin-ligase complex SCF^TIR1^/ABF (SKP1–CULLIN–F-box), recruits them for degradation and releases ARFs from repression [15,17]. The other auxin-related target genes are small auxin up regulated genes (SAURs), and auxin responsive genes. *AtDwarfs* (*DWFs*) are a critical BR synthetic genes in BR-mediated signaling pathway [18]. Also, calcium signaling is reported to play a critical role in BR-signaling pathway through DWF1, a CaM-binding enzyme in the BR biosynthetic pathway [19]. Endogenous BRs are recognized by LRR receptor proteins or receptor-like proteins such as BRI1/DWF2 (Brassinosteroid-Insensitive 1/AtDwarf 2) and BRLs (Brassinosteroid-Insensitive like) genes [20,21,22,23,24]. *Brassinazole resistant 1* (*BRZ1*) and its homologs constitute a family of DNA-binding transcription factors that positively regulate the expression of BR-induced genes and plant growth [25]. Changes in the expression of these genes have been shown to have significant impact on plant growth.

Following pathogen attack, AtSR1 was shown to be degraded through ubiquitin-mediated proteolytic pathway [1,26]. Due to the degradation of AtSR1 triggered by pathogen infection, the defense-related genes *NDR1* and *EDS1* [27,28,29] are de-repressed, and this will subsequently activate the expression of *ICS1* [30], a SA synthetic gene which contributes to most of the pathogen-induced SA biosynthesis. The elevated SA is recognized by the SA receptor, nonexpresser of PR genes1 (NPR1) [31,32]. SA is required for the interaction of NPR1, TGAs, and NIMIN1 to form a temporal protein complex which binds to the promotor of defense-related genes including *PR1* to induce their expression and to establish plant immune responses [33,34]. In addition to SA-mediated signaling pathway, AtSR1 is also reported to be involved in jasmonate (JA)-mediated signaling pathway that is known to respond to herbivore-induced stress [35].

Our knowledge about the role of AtSR1 as transcription factor in regulating the trade-offs between plant growth and immunity is limited. In this study, we performed a global gene expression profile using Affymetrix microarrays to reveal the role of AtSR1-regulated defense- and growth-related genes, in the absence of pathogen and during pathogen infection. Results described here will help to determine whether the changes in growth in *atsr1* mutant are associated with changes in auxin and/or BR responses signaling pathway. Molecular approaches were used to monitor the regulatory relationships of these auxins and BR-related genes by AtSR1 to investigate whether AtSR1 balances plant growth and immunity when the plant is under attack by pathogens.

## 2. Results

### 2.1. AtSR1 Is a Negative Regulator of Immune Response and a Putative Positive Regulator of Growth and Development in Plants

**Figure 1:**
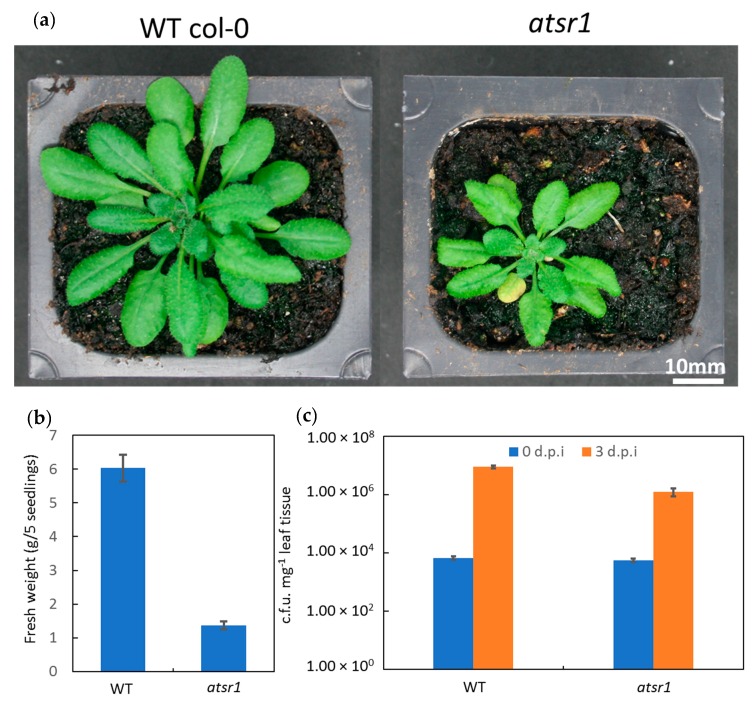
*Arabidopsis* Signal Responsive 1 (AtSR1) is involved in plant immune response as well as growth and development. (**a**) Phenotypic comparison. Five-week-old wild-type (WT) and the *atsr1* loss-of-function plants were grown at 20–22 °C under a 12 h photoperiod. Scale bar represents 10 mm. (**b**) Growth comparison. Fresh weight of rosettes was calculated from five-week-old WT and the *atsr1* loss-of function mutant were grown at 20–22 °C under a 12 h photoperiod. Values are means ±SD (*n* = 5). (**c**) Disease resistance test for WT and *atsr1*. Rosette leaves of five-week-old *Arabidopsis* were infiltrated with *Pst* DC3000 (OD_600_ = 0.001), and the number of colony-forming units (cfu) was calculated at zero (1 h after inoculation) and three days post-inoculation (dpi). Values are means ± SD (*n* = 5).

At 20–22 °C (12 h light photoperiods), the *atsr1* mutants displayed typical temperature-dependent autoimmune phenotypes, including spontaneous lesions in their leaves and semi-dwarfism (Figure 1a). Also, fresh weight (g/5 seedlings) of five-week-old *atsr1* mutant plants were lower compared to that of wild-type (Figure 1a,b). In addition, we infiltrated leaves from four- or five-week-old plants of wild-type and *atsr1* mutant with the DC3000 virulent strain of *P. syringae* pv. Tomato; and the infected leaves were collected after zero (1 h after inoculation) or three days after the infection for disease resistance test. At zero days, both wild-type and *atsr1* mutants hosted similar numbers of the DC3000 bacteria. However, after 3threedays, wild-type plants hosted 8.93 × 10^6^ colony forming units (cfu) mg^−1^ of the pathogen in their leaf tissue, whereas *atsr1* plants hosted only 1.24 × 10^6^ cfu mg^−1^ in their leaf tissue (Figure 1c). The differences in growth and resistance to pathogen infection between WT and *atsr1* mutant plants imply that AtSR1 plays a key role in balancing plant immunity and growth, acting as a negative regulator in plant immune response and a positive regulator of plant growth. We then decided to test the fold change of defense- and growth-related genes in *atsr1* mutant plants as compared to that in WT.

### 2.2. AtSR1-Mediated Transcriptome Landscape in Plant Growth and Immune Responses

RNA samples isolated from WT and *atsr1* mutant plants were subjected to transcriptomic analysis using *Arabidopsis thaliana* hybridization1 (ATH1) microarray chip (two independent biological repeats were performed for each genotype and treatment combination). Gene expression data from the *atsr1* mutant plants were compared to that of WT to determine the genes that are elevated or repressed.

**Figure 2:**
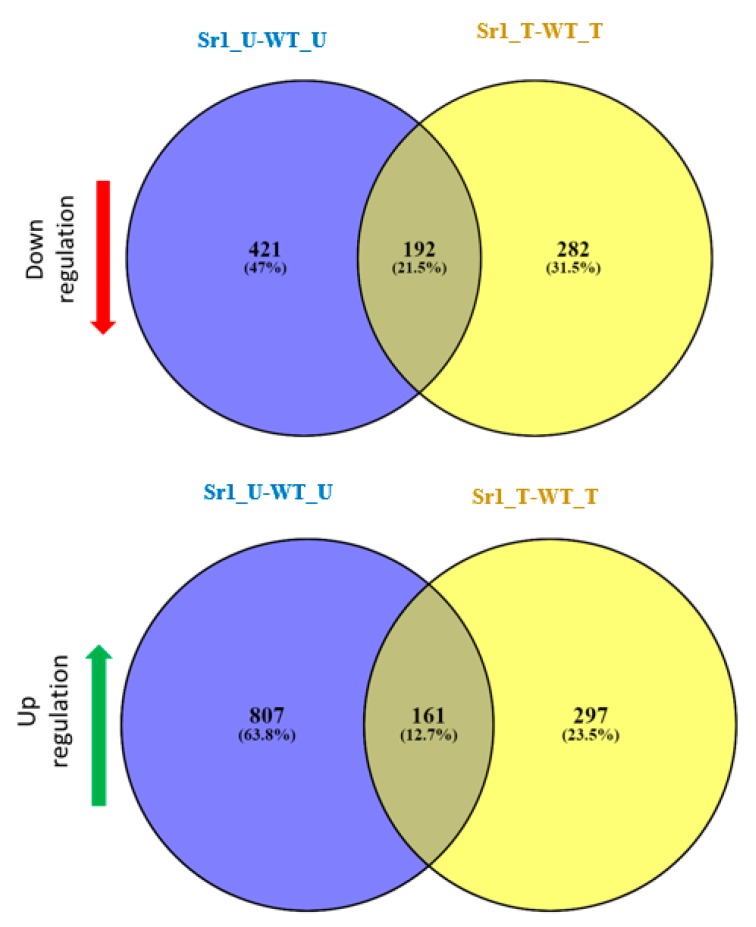
Comparative analyses of transcriptomes in WT and *atsr1*. Venn diagram of a double-sided comparison of microarray expression data revealing the circles which represent total number and arrows which represent the number of differentially expressed genes (DEGs) between the genotype test or pathogen inoculation. 192 genes were observed that are down-regulated with a fold change (FC) ≤ −2 and *p*-value < 0.05 in both of the tests, and 161 genes were observed to be up-regulated with an FC ≥ 2 and *p*-value < 0.05 in both of the tests. WT_U: WT untreated; WT_T: WT treated with pathogen infection for 24 h after infiltration with *Pst* DC3000; Sr1_U: sr1 untreated; Sr1_T: Sr1 treated with pathogen infection for 24 h after infiltration with *Pst* DC3000.

To identify the genes regulated by AtSR1, we compared the whole transcriptomes of the WT (Col-0) and *atsr1* mutant plant with or without pathogen inoculation. We compared the transcriptomes in the absence of a pathogen treatment and our results revealed that 1581 genes were differentially expressed in Sr1_U (*atsr1* un-treated) as compared to WT_U (WT un-treated, Figure 2). Among them, 613 genes (39%) were observed to be down-regulated, while 968 genes (61%) were observed to be up-regulated in *atsr1*. To identify the genes regulated by AtSR1 during plant response to pathogen attack, we infiltrated the rosette leaves of five-week-old WT (WT_T, WT treated) and *atsr1* (Sr1_T, Sr1 treated) plants with *Pst* DC3000 (OD_600_ = 0.001) and leaf samples were harvested at 24 hours post inoculation (hpi). RNA from these samples was extracted for whole transcriptome analysis (Figure 2). 932 genes were observed to be differentially expressed in *atsr1_*T as compared to WT_T. In this group, 474 (~50%) genes were observed to be down-regulated in *atsr1*, while 458 genes (~50%) were observed to be up-regulated. Comparing differentially expressed genes (DEGs) from Sr1_U vs. WT_U and that from Sr1_T vs. WT_T, we found that the expression of 192 of these genes were down-regulated and 161 genes were up-regulated in both of the tests.

**Figure 3:**
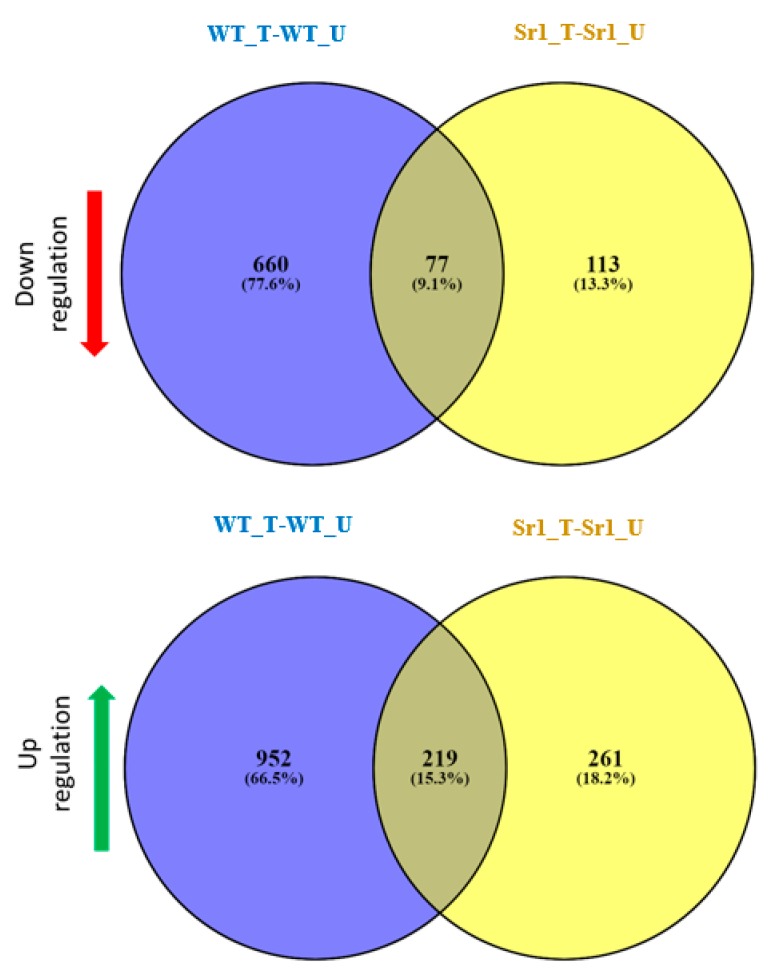
Comparative transcriptome analysis of WT and *atsr1*. Venn diagram of a double-sided comparison of microarray expression data revealing the circles that represent total number and arrows that represent the number of differentially expressed genes (DEGs) between the genotype test or pathogen inoculation. 77 genes were observed to be down-regulated with a FC ≤ −2 and *p*-value < 0.05. 219 genes were observed that are up-regulated with a FC ≥ 2 and *p*-value < 0.05. WT_U: WT untreated; WT_T: WT treated with pathogen infection for 24 h after infiltration with *Pst* DC3000; sr1_U: sr1 untreated; sr1_T: sr1 treated with pathogen infection for 24 h after infiltration with *Pst* DC3000.

In addition, in order to determine how pathogen infection induces transcriptional reprogramming in plants, we first compared the gene expression profiles in WT 24 h post inoculation to that without inoculation. As shown in Figure 3, 1908 genes were identified to be differentially expressed after pathogen attack in WT. Among them, 1171 genes (61%) were observed to be induced, while 737 genes (39%) were observed to be repressed by *Pst* DC3000 at concentration OD_600_ = 0.001. We also compared the gene expression profiles 24 h post inoculation to that without inoculation in *atsr1*. One hundred and ninety (28%) of the genes were observed to be down-regulated, while 480 genes (72%) were observed to be up-regulated in *atsr1* mutant plants (Figure 3). In addition, comparing DEGs from WT_T vs. WT_U and that from Sr1_T vs. Sr1_U, we found that the expression of only 77 (9.1%) of these genes were down-regulated and 219 genes were up-regulated in both of the tests.

### 2.3. AtSR1 Negatively Regulates Plant Immune Pathway in the Absence of Pathogen

**Figure 4:**
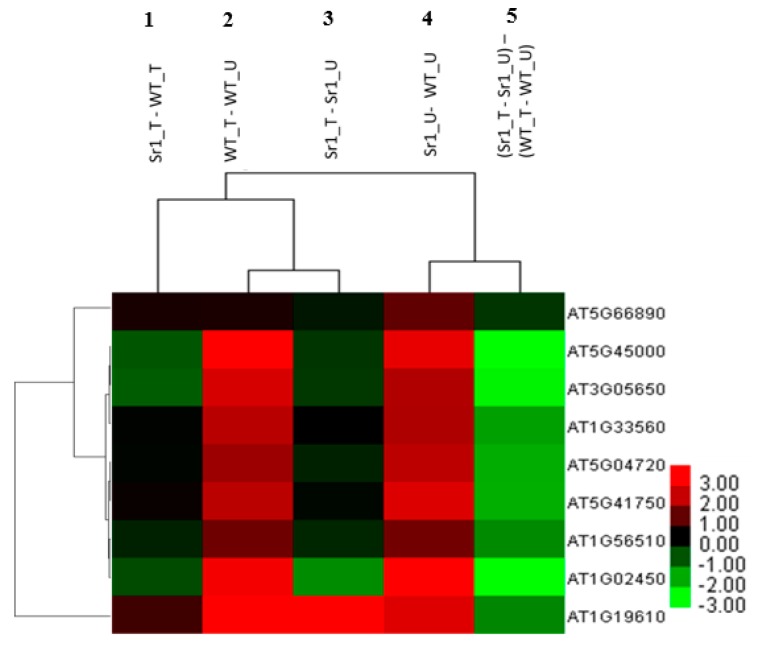
Hierarchical cluster analysis and heatmap indicating expression of defense-related DEGs between the genotype test and pathogen inoculation. The fold changes in gene expressions that increased and decreased in abundance are indicated in red and green, respectively. The intensity of colors increased as the difference in abundance increased. WT_U: absence of pathogen in WT; WT_T: 24 h after infiltration with *Pst* DC3000 in WT; Sr1_U: absence of pathogen in *atsr1* mutants; Sr1_T: 24 h after infiltration with *Pst* DC3000 in *atsr1* mutants.

AtSR1 has been reported to negatively regulate plant immune response by suppressing the expression of defense-related genes, *EDS1* and *NDR1*, which are documented to be required for the activation of SA pathway through ICS1, a key enzyme required for SA biosynthesis [36,37]. We analyzed whether AtSR1 is involved in the regulation of Nucleotide-Binding Leucine Rich Repeat (NLR) genes which are involved in the very early stage of pathogen perception and up-stream to EDS1 and NDR1 regulated steps [38,39,40]. The microarray data revealed that TIR-NBS-LRR proteins (At5g45000, At5g41750 and At1g56510) were induced in *atsr1* mutant plants as compared to WT plants. Similarly, the transcriptional expression of CC-NBS-LRR protein, At5g66890, was induced in *atsr1* mutants. Recent observations revealed that truncated NLR proteins play a key role in NLRs-mediated plant immunity, also known as effector triggered immunity (ETI). Hence, we also tested the expression of NBS-LRR protein, ACTIVATED DISEASE RESISTANCE 1 (ADR1), and ADR-like 2 (ADR1-L2) (Figure 4, lane 4) [41,42,43,44]. There is a significant induction of these genes in *atsr1*. These results suggest that AtSR1 repressed the gene expression of multiple NLRs.

We then tested the expression of defense-related genes which were involved in the down-stream regulation of the SA-mediated signaling pathway. It has been documented that NPR1 acts as a SA receptor and plays a fundamental role in the down-stream regulation of the SA pathway [45,46]. Interestingly, we found the *NPR1* expression was also induced in *atsr1*. In addition, we found that the transcription of NIM1-INTERACTING 1 (NIMIN1) is also induced in *atsr1* mutant plants (Figure 4, lane 4). During pathogen attack, NIMIN1, together with NPR1 and TGAs, induced the expression of PR1 through a temporal complex to bind to the PR1 promoter.

In addition to SA pathways, we also determined the transcriptional expression of other genes which were involved in immune response. Plant defensin (PDF) 1.4, belongs to the PDF family which is known to be involved in jasmonic acid (JA)-regulated immune pathway [47,48]. A 2.25-fold change in log 2 of PDF 1.4 expression was observed in *atsr1* mutants as compared with that in WT. Furthermore, pathogen associated molecular patterns (PAMPs) are known to be recognized by pattern recognition receptors (PRRs), such as Toll-like receptors (TLRs) and nucleotide-binding oligomerization domain-like receptors, which are known to be the first step of plant innate immune response, also known as PAMP-triggered immunity (PTI). We also observed the receptor-like protein 32 (RLP32), a PAMPs receptor-like kinase [49,50], was induced in *atsr1* mutants (Figure 4, lane 4). Obviously, AtSR1 regulates many steps in plant immunity, from NLRs- and PRRs-mediated pathogen detection, to activation of SA biosynthesis and SA signal perception, as well as JA-dependent plant immune responses.

### 2.4. AtSR1-Regulated Immune Pathway Response to Pathogen Attack

We analyzed how AtSR1-mediated defense-related genes are expressed during pathogen infection. We infiltrated rosette leaves of WT and *atsr1* mutants with *P. syringe.* Samples were harvested for RNA extraction 24 h after infiltration and global gene expression analysis was performed using Affymetrix ATH1 microarrays. As we expected, the fold change in the expression of defense-related genes mentioned above in the comparison of WT_T vs. WT_U (Figure 4, lane 2) was almost the same as that in the comparison of Sr1_U vs. WT_U (Figure 4, lane 4). This result indicates that AtSR1 regulates a distinct group of signaling pathways responding to pathogen attack.

Comparisons of sr1_T vs. Sr1_U (Figure 4, lane 3) and WT_T vs. WT_U (Figure 4, lane 2) (or Sr1_U against WT_U, Figure 4, lane 4) revealed the gene expression triggered by pathogen in *atsr1* is different from that in WT. Only one gene, PDF 1.4, was induced by pathogens in both WT and *atsr1*. The expression of the other 8 defense-related genes was downregulated in *atsr1* after pathogen treatment as compared to that in WT. Similar results were observed when comparing Sr1_T and WT_T (Figure 4, lane 1). Furthermore, in the comparison of Sr1_T vs. sr1_U against WT_T vs. WT_U (Figure 4, lane 5), all of the defense-related genes induced in WT were found to be repressed in *sr1* mutant after pathogen attack. These results imply that AtSR1-regulated plant immune pathway responds to pathogen attack not through the enhancement of basal defense, but through rapid establishment of inducible immune reactions.

### 2.5. AtSR1 Positively Regulates Auxin- and BRs-Mediated Plant growth and Development in the Absence of Pathogen

**Figure 5:**
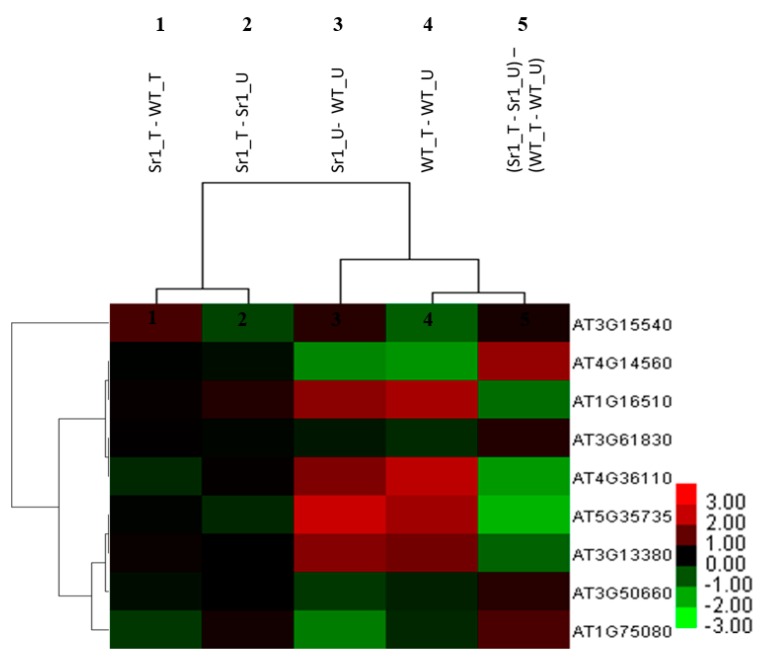
Hierarchical cluster analysis and heatmap indicating expression of growth-related DEGs between the genotype test and pathogen inoculation. The fold changes in gene expressions that increased and decreased in abundance are indicated in red and green, respectively. The intensity of colors increased as the difference in abundance increased. WT_U: absence of pathogen in WT; WT_T: 24 h after infiltration with *Pst* DC3000 in WT; Sr1_U: absence of pathogen in *atsr1* mutants; Sr1_T: 24 h after infiltration with *Pst* DC3000 in *atsr1* mutants.

We studied growth-related genes affected by AtSR1. Auxin and BRs are two endogenous growth-promoting phytohormones [13,17,22]. Two genes positively correlated with plant growth, ARF18 and DWF4 in auxin- and BR-mediated pathway, respectively, were identified to be suppressed in *atsr1* mutants as compared to that in WT. In addition, four auxin-related genes in the auxin-mediated pathway were induced in *atsr1*, including At5g35735 (auxin-responsive family protein but function is unknown), INDOLE-3-ACETIC ACID INDUCIBLE 19 (IAA19) and two Small Auxin Upregulated (SAUR) genes, *SAUR41* and *SAUR9*; while *IAA1* is repressed in *atsr1*. BRASSINAZOLE-RESISTANT 1 (BZR1) was documented as a transcription factor which interacts with E box sequences (CANNTG) mediating responses to BR signals during plant growth and development. Similar to DWF4, the expression of BZR1 was also repressed in *atsr1*, while the expression of BRI1-LIKE 3 (BRL3), a plasma membrane-located brassinosteroid receptor protein, was induced in *atsr1* (Figure 5, lane 3). These observations suggest that AtSR1 is involved in plant growth through auxin- and BR-mediated signaling pathways.

### 2.6. AtSR1-Regulates Plant Growth-Related Pathway in Response to Pathogen Attack

To determine the role of AtSR1 in balancing growth and immunity during pathogen infection, we tested the growth-related gene expression in response to pathogen attack. As expected, the expression changes of growth-related genes (Figure 5) in comparison between WT_T and WT_U (Figure 5, lane 4) was almost the same as that in comparison between Sr1_U and WT_U (Figure 5, lane 3). Only one exception, IAA19 was repressed during pathogen infection in WT. These results indicate that, while regulating the immune related genes, AtSR1 also coordinates the expression of growth related genes in the auxin- and BR-mediated signaling pathways during pathogen attack.

The results of comparisons of Sr1_T vs. Sr1_U (Figure 5, lane 2), WT_T vs. WT_U (Figure 5, lane 4) and Sr1_U vs. WT_U (Figure 5, lane 3) revealed that the change in expression of growth related genes triggered by pathogen in *atsr1* is also similar to that in WT. Only three genes (At5g35735, BRL3, and DFW4) displayed differential expression in these comparisons. However, in Sr1_T vs. Sr1_U against WT_T vs. WT_U (Figure 5, lane 5), most of the growth-related genes displayed different gene expressions during pathogen attack. The only exception was again IAA19 (Figure 5). These results indicate that AtSR1 regulates plant growth-related pathways upon pathogen attack by reprogramming growth-related genes.

### 2.7. AtSR1 Could Bind to the CG-Box Motif in the Promotor of Target Genes

We analyzed the promotors of defense- and growth-related genes mentioned above, since these candidate genes were identified by the combined use of high throughput expression profile (microarray analysis) and in silica analyses. A typical CG-box with “CGCG” core sequence was found in the promoters, indicating a possible direct regulation of these genes by AtSR1.

**Figure 6:**
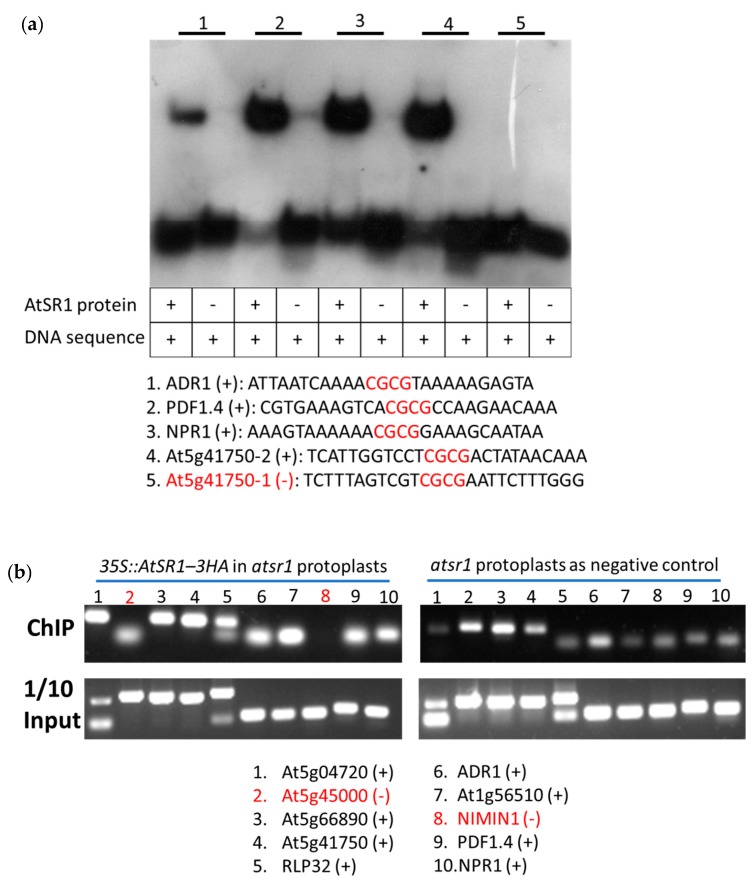
AtSR1 binding to the promoters of defense-related genes. (**a**) Promoter fragments of defense-related genes and purified recombinant AtSR1 protein were used in EMSA assay. At5g41750-1 in red indicates negative result (−). (**b**) The ChIP’ed DNA was amplified with primers flanking the CGCG box in the promoters of defense-related genes. At5g45000 and NIMIN1 in red indicates negative results (−). See also Table 1.

**Figure 7:**
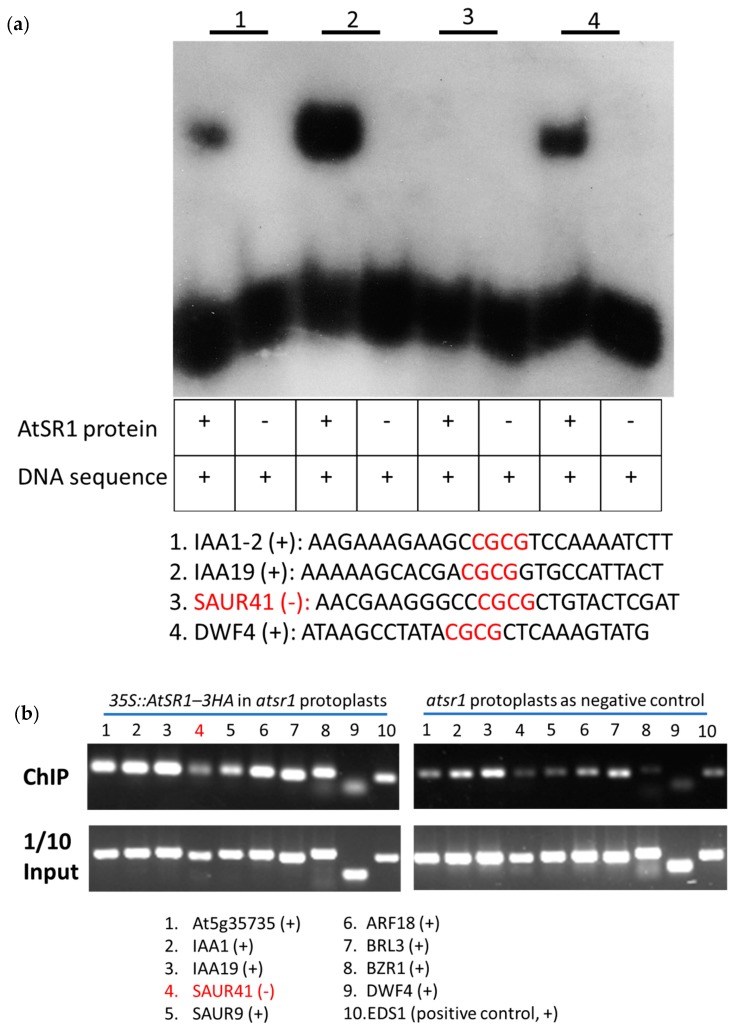
AtSR1 binding to the promoters of growth-related genes. (**a**) Promoter fragments of growth-related genes and purified recombinant AtSR1 protein were used in EMSA assay. (**b**) The ChIP‘ed DNA was amplified with primers flanking the CGCG box in the promoters of growth-related genes. SAUR41 in red indicates negative result (−). See also Table 2.

We synthesized short double-stranded DNA fragments corresponding to a short promoter section (26 bp) of DNA containing the CGCG-box in the candidate genes. Using electrophoretic mobility shift assay (EMSA), we verified their interaction with the recombinant AtSR1 protein (1–153 amino) covering the intact DNA-binding domain in vitro (Figure 6a or Figure 7a and Table 1 and Table 2). Although the binding signals varied, our results revealed that at least one of the promoter fragment from the tested genes involved in defense- (8 out of 10) or growth (7 out of 9)-related genes interacted with the recombinant AtSR1 protein (Figure 6a, Figure 7a and S1–S4).

Furthermore, we determined that full-length AtSR1 protein interacted with the promotors of the genes mentioned above *in planta*, using chromatin immunoprecipitation (ChIP) coupled with PCR analysis. AtSR1 protein with 3HA tag was transiently expressed in the protoplasts isolated from *atsr1* mutant plants. ChIP’ed DNA was collected using anti-HA antibody. Similar to the EMSA data, the ChIP-PCR data revealed that full-length AtSR1 protein interacted with eight out of the 10 tested defense-related genes and eight out of nine of the tested growth-related genes, respectively (Figure 6a and Figure 7a, Table 1 and Table 2). These results suggest that AtSR1 acts as a transcription factor balancing immunity and growth by regulating target genes which contains the “CGCG” containing CG-box(es) in their promotor.

## 3. Discussion

Using Affymetrix microarrays, we performed a comprehensive analysis of the defense- and growth-related genes triggered by *Pst* DC3000 in WT and *astr1*, which indicated a role for AtSR1 in balancing plant immunity and growth in response to pathogen challenges, but also in the absence of pathogens. *atsr1* mutant plants displayed enhanced disease resistance and dwarfism, and this pleiotropic phenotype of *atsr1* mutant could be completely rescued by ectopic expression of AtSR1 CDS driven by 35S promoter [4]. The microarray data suggested AtSR1 regulated the transcriptional expression of immune-related genes which are involved in PTI and ETI, as components in JA- or SA-mediated signaling pathway (Figure 2 and Figure 3 and Tables S1–S5). Results from this study and previous reports indicate that AtSR1 actively regulates several steps of the SA pathway, from activation of SA synthesis to SA signal perception [4,7,51,52,53]. At the same time, AtSR1 may also regulate plant growth through auxin- and BR-related pathway.

How AtSR1 regulates the plant immune response during pathogen attack is still not fully understood. A previous report has revealed that the pathogen-triggered AtSR1 degradation mechanism is achieved through the ubiquitination of AtSR1 by SR1IP1 (a substrate adaptor for CUL3-based E3 ligase) [26,54]. This could explain the similarity of DEGs between WT_T vs. WT_U and sr1_U vs. WT_U (Figure 4 and Figure 6). Comparing *atsr1* to WT in the absence of pathogens (sr1_U vs. WT_U), AtSR1 protein was missing in the *atsr1* null mutant; while in WT during pathogen attack (WT_T vs. WT_U), AtSR1 protein was degraded after pathogen infection (Figure 4 and Figure 6). Therefore, the gene expression profiles are similar in these two situations.

In addition, a previous observation revealed that, although the maximum level of expressions of PR1 were similar in wild-type and *atsr1* plants after inoculation of pathogen, the expression of PR1 was induce 6 h after inoculation in *atsr1* plants, while the induction of PR1 did not start until 24 h after inoculation in WT [4]. Also, the accumulation of SA triggered by pathogen was faster in *atsr1* plants as compared to that in WT. These observations are consistent with our microarray data comparing the expression profile of Sr1_T vs. Sr1_U and WT_T vs. WT_U, indicating that AtSR1-regulated plant immune response to pathogen attack is through the establishment of a rapid and appropriate immune response. In other words, the degradation of AtSR1 upon pathogen attack initiates a faster reprogramming of transcriptional expression of defense-related genes or rapid production of SA, but not through the enhancement of basal defense levels. In this way, the activation of immune response through the turnover of AtSR1 specifically after pathogen infection will incur less energy loss which could otherwise be used in growth and development.

In addition, using both EMSA and ChIP-PCR we observed that AtSR1 protein recognizes and interacts with promotors of most of its putative target genes both in vitro and in vivo. However, AtSR1 could not recognize the promotors of *SAUR41* and *unknown gene (At5g45000)* in either EMSA or ChIP-PCR, although their promotors include the typical AtSR1-recogized “CGCG” motif. This suggests that the flank sequence of VCGCGB might have a possible impact on the interaction between AtSR1 protein and its VCGCGB target sequence. Also, ChIP-PCR assay is prone to verify more AtSR1-target gene recognition events as compared to EMSA assay, such as *BZR1* promotor. A reasonable explanation is that AtSR1 protein would recognize other motif(s), besides CGCG, such as CGTG or CACG [55,56].

We also confirmed AtSR1 is able to interact with most of the promotors of genes in both defense- and growth-related signaling pathways (Figure 5 and Figure 7). However, the mechanisms of how AtSR1 up-regulates some genes while down-regulates others are still not clear. Future studies about identification of other TFs or co-TFs binding to AtSR1 or other AtSR1-targeted motifs in promoter regions would elucidate this difference.

## 4. Materials and Methods

### 4.1. Plant Material and Growth

The *Arabidopsis* lines used in this study are wild-type (*WT*) Columbia (Col-0) and loss-of function *atsr1* lines (Salk_001152) obtained from an earlier project [4]. Seeds were surface sterilized with 1/3 diluted bleach and germinated on half-strength MS medium (Caisson Laboratories Inc., Smithfield, UT, USA) [57]. One-week-old seedlings were transferred to pots containing soil mix. Plants were maintained in a growth chamber under a 12-h photoperiod at 20–22 °C and were watered as needed.

### 4.2. Plant Pathogen Infection and Disease Resistance Assays

*Pseudomonas syringae* pv. tomato DC3000 was cultured in King’s B medium and inoculation was performed as previously described [4]. Briefly, leaves of 5-week-old plants grown at 20–22 °C with 12-h photoperiod were infiltrated with *Pst* DC3000 at OD_600_ = 0.001 in 10 mM MgCl_2_ using 1 mL needleless syringe for disease resistance test and microarray analysis. Each disease resistance result is the average of five replicates; the results are presented as mean ± s.d.

### 4.3. Total RNA Extraction and mRNA Isolation

Total RNA for microarray was isolated from 2–4 g leaf material of five-week-old *Arabidopsis* seedlings. Control and infected seedlings of different genotypes were collected and flash frozen in liquid nitrogen (N_2_). The frozen tissues were ground to powder in 1.5 mL microfuge. Total RNA was prepared using TRIzol Reagent (Invitrogen, Carlsbad, CA, USA) followed by DNase-I (Roche) treatment. mRNA was isolated from 20 μg total RNA (Qiagen Oligotax mRNA Mini Kit, Hilden, Germany), as recommended by the manufacturer. The isolated mRNA as the template, cDNA libraries were synthesized with a mixed primer with an oligo (dT) primer and random hexadeoxynucleotides primer. The cDNA sample was sent to the WSU Molecular Biology and Genomics Core (originally named the Laboratory for Biotechnology and Bioanalysis) for further Affymetrix Microarrays analysis.

### 4.4. Differential Expression Genes Analysis of Microarray Data

The Affymetrix Microarray data were obtained from Molecular Biology and Genomics Core. Robin/RobiNA tool was used for differential expression genes analysis [58,59]. The raw data of “.CEL” files were loaded into the Affymetrix GeneChip microarray experiment in Robin/RobiNA. Box and MA plot together with scatterplot were run as microarrays quality check tools. Configure grouping was described above. The raw data were normalized by RMA with hierarchical multiple testing strategy. Transcripts expression of microarray identifier/elements were cut off with fold change (FC) ≤ −2 or FC ≥ 2 and *p*-value < 0.05. The *p*-value correction was set as BH. Microarray identifier/elements were annotated by using Arabidopsis_thaliana_AFFY_ATH1_TAIR10_Aug2012.m02. One hundred percent of the identifier was annotated by using mapping file, and the total No. of mapped microarray identifier is 22810. The results were viewed in MapMan tool for further analysis [60,61].

### 4.5. Clustering of Microarray Data

The normalized data from Robin/RobiNA were loaded into Gene Cluster 3.0 tool for Hierarchical cluster analysis [62]. First, the normalized data were filtered with 80%, SD (Gene Vector) 2.0, observation with abs (Val) ≥2 and MaxVal-MinVal ≥2. The filtered data was used for hierarchical cluster analysis with gene cluster (calculated weight). The similarity metric was set to Correlation (centered). Complete linkage was used as clustering method. The results were exported as “.cdt” (clustered data table) files. The “.cdt” files were viewed in Java Treeview tool, and the results are described above.

### 4.6. EMSA for Confirmation of the Interaction between AtSR1 Protein and Short DNA for the Promotor of Target Genes

The *E. coli* strain BL21(DE3)/pLysS carrying the above pET32a-derived plasmids for expression of recombinant proteins of the wild-type versions of AtSR1 were obtained from a previous project. The *E. coli* strain was cultured in Super Optimal broth with Catabolite repression (SOC) media for 3 h with the concentration of OD_600_ = 0.5. Then, Isopropyl β-D-1-thiogalactopyranoside (IPTG) was added with a final concentration of 0.5 mM IPTG for 3 h. 6His-tagged recombinant proteins were purified using Ni-NTA agarose affinity beads (Qiagen) as described by the manufacturer [63,64]. Recombinant AtSR1 covering CG-1 domain was used for EMSA to detect its interaction with the promoter fragments of target genes.

### 4.7. Mesophyll Protoplast Isolation and Transfection for Transient AtSR1 Expression

We optimized previous methods as follows [65,66,67,68]. Briefly, mesophyll protoplasts were isolated from two or three-weeks old *atsr1* mutant plants, using the Tape-*Arabidopsis* Sandwich method. The isolated protoplasts were filtered through 70-μm mesh into 50 mL tubes and then were harvested by centrifugation at 200× *g* for 3 min at 4 °C. The isolated protoplasts were washed twice by W5 solution (154 mM NaCl, 125 mM CaCl_2_, 5 mM KCl, 5 mM glucose, and 2 mM MES at pH 5.8). Protoplasts were counted by hemocytometer under a light microscope. The protoplasts were then centrifuged at 200 g for 3 min at 4 °C and resuspended in MMg solution (0.4 M mannitol, 15 mM MgCl_2_, and 4 mM MES, pH 5.8) to a final concentration of 2 × 10^8^ cells/mL. 40 μg of plasmid DNA (*35S::AtSR1–3HA*) were mixed into protoplasts in MMg solution. An equal volume of a freshly-prepared solution of 40% (*w*/*v*) 4000 Polyethylene Glycol (PEG) was gently added, and then the mixed solution was incubated at room temperature for 5 min. After incubation, the mixture was washed three times by W5 solution. Finally, the protoplasts were placed in 1 mL of W5 solution and were incubated in 6-well plates coated with 1% bovine serum albumin (BSA) at 22 °C for 15–20 h in growth champers with light.

### 4.8. ChIP (Chromatin Immunoprecipitation)-PCR

The transfected protoplasts were harvested by centrifugation at 200× *g* for 3 min at 4 °C. Protoplasts were resuspended in autoclaved phosphate buffered saline (PBS) solution (pH 7.5) containing 1% formaldehyde and vacuumed for 10 min. Samples were then quenched by 2 M of glycine that is sucked into the protoplasts by vacuum for another 5 min [69]. The DNA of samples was sheared with a sonicator for 10 min on ice: the output power was set at “10”, and the sample was treated for 10 s by running the “ON cycle” and then 50 s in “OFF cycle.” The chromatin samples were pre-cleared with Protein A agarose beads. Pre-cleared chromatin was immuno-precipitated with Protein A agarose beads and HA-antibody overnight. The bound agarose beads were washed by PBS buffer, low-salt buffer, high-salt buffer, LiCl buffer, and TE buffer twice. The DNA was eluted in SDS-NaCl buffer at 65 °C for 15 mins. The IP-DNA was cleaned by PCR Purification Kit (Qiagen) and then used for PCR.

## Figures and Tables

**Table 1:** Putative AtSR1 target genes related to plant immune response.

Ratio	Direction	AGI Code	Functional Description	ChIP/SR1	EMSA/SR1
1.90	↑	At5g04720	Encodes a member of the ADR1 family NB-LRR immune receptors	+	+
2.36	↑	At5g45000	Disease resistance protein (TIR-NBS-LRR class) family	−	−
0.99	↑	At5g66890	Disease resistance protein (CC-NBS-LRR class) family	+	+
2.24	↑	At5g41750	Disease resistance protein (TIR-NBS-LRR class) family	+	−,+,+
1.77	↑	At3g05650	receptor like protein 32 (RLP32); FUNCTIONS IN: kinase activity	+	+
1.77	↑	At1g33560	ACTIVATED DISEASE RESISTANCE 1, ADR1.	+	+
1.16	↑	At1g56510	TIR-NB-LRR protein confers resistance to Albugo candidate	+	−
3.23	↑	At1g02450	NIMIN1 modulates PR gene expression with other TF(s)	−	−,+,−,−
2.25	↑	At1g19610	LCR78, LOW-MOLECULAR-WEIGHT CYSTEINE-RICH 78, PDF1.4	+	+
1.00	↑	AT1G64280	NPR1 (NONEXPRESSER OF PR GENES 1); Co-transcription factor	+	+

“Ratio” = fold expression in *sr1* as compared to WT; “Direction” = “↑” (increase) or “↓” (decrease) in expression; “ChIP/SR1” = chromatin immunoprecipitation (ChIP) assay of promoter of each gene interacting with full-length AtSR1 protein in planta; “EMSA/SR1” = electrophoretic mobility shift assay (EMSA) assay of each cg-box containing fragment interacting with cg1 domain of SR1. As a reference, the ratio of expression level (log2) of PDF1.4 (At1g19610) in *atsr1* vs. WT is 2.25, direction is up-regulation.

**Table 2:** Putative AtSR1 target genes related to actions of growth hormones.

Ratio	Direction	AGI Code	Functional Description	ChIP/SR1	EMSA/SR1
2.04	↑	At5g35735	auxin-responsive family protein with unknown function	+	+,−
1.57	↓	At4g14560	auxin-responsive protein/indoleacetic acid-induced protein 1 (IAA1)	+	−,+
0.44	↑	At3g15540	auxin-responsive protein/indoleacetic acid-induced protein 19 (IAA19)	+	+
1.40	↑	At1g16510	SAUR41, small auxin up-regulated protein 41	−	−
1.27	↑	At4g36110	SAUR9, small auxin up-regulated protein 9	+	+
0.34	↓	At3g61830	ARF18, auxin-responsive factor gene family, AUX/IAA-related	+	+,+
1.34	↑	At3g13380	An LRR receptor-like kinase similar to BRI (BRL3)	+	+
1.47	↓	At1g75080	brassinosteroid signaling positive regulator (BZR1)	+	-
0.69	↓	At3g50660	steroid 22-alpha-hydroxylase (CYP90B1) (DWF4)	+	+

“Ratio” = fold expression in *sr1* as compared with WT; “Direction” = “↑” (increase) or “↓” (decrease) in expression; “ChIP/SR1” = chromatin immunoprecipitation (ChIP) assay of promoter of each gene interacting with full-length AtSR1 protein in planta; “EMSA/SR1” = electrophoretic mobility shift assay (EMSA) assay of each cg-box containing fragment interacting with cg1 domain of SR1. As a reference, the ratio of expression level (log2) of IAA1 (At4g14560) in *atsr1* vs. WT is 1.57, direction is down-regulation.

## Supplementary Materials

Supplementary materials can be found at http://www.mdpi.com/1422-0067/19/6/1764/s1.
