# Supplementary material for: Ca2+/Calmodulin-Dependent AtSR1/CAMTA3 Plays Critical Roles in Balancing Plant Growth and Immunity
Source: Int J Mol Sci. 2018 Jun 14;19(6):1764. doi: 10.3390/ijms19061764 (PMC6032152; doi:10.3390/ijms19061764)
Supplement: Supplementary file 1 [file ijms-19-01764-s001.zip › IJMS S Table 6 and 7.pdf]

Supplementary Table 6. List of primers for ChIP-PCR for defense related genes

Gene Name/ID	Forward primer	Reverse primer
At5g04720	GCTATGGTGCCACTTCTAT	CTACGGAGACCCAATAAAGA
At5g45000	TTTGCTTTGCAGGCTTAC	TCTGGTATAGTCACTTTCTAAA
At5g66890	GAACAGAGTTATTCACCAAC	TTGACCGACTCTTTCTTTG
At5g41750	TCAGTTCCAGTTACATTCCA	GAGAAACCCTTTACGGACAT
RLP32	GCGTTGGTTCTTCAAGGTGTT	CGTCTTCAATTCCGTGATTTAGG
ADR1	GGAGCTGGACCAACACTA	GGATTAGGGACTCGAACTTT
At1g56510	GTTATGTACGCTTCATTACG	AGACATTGTATCTCCAGTTG
NIMIN1	AATAAATCGTCCCGCGATATACC	ATCTCATATTGTCGGCCACTTG
PDF1.4	GCATAGGCGAAGAACAAGAAGTA	AGCACATGACTCATCTCATCTCA
NPR1	AATATGTGATGCTATTGAGTTATAGAGA	GCCTATGAATACAACAACGTAAGAT

Supplementary Table 7. List of primers for ChIP-PCR for growth related genes

Gene Name/ID	Forward primer	Reverse primer
At5g35735	ACGAGAGGGAAGGGAATTGAA	GCCTACATACATATCGCCTCAG
IAA1	GTGTGGTAGAAGGACGAAGGT	GGAGCATGAGGAGGCAAGTT
IAA19	GACCACCGCATCCTCAGTT	GAACTTTCTTCTCCTACACTTCTCT
SAUR41	TCTTGTCGGGCAGATAAACTAT	GAAGATTACACGACCAAGAAGTC
SAUR9	TTGAAGAGATCATATAAGATAATGTACG	TTTGGATGATTTGTGACTAGGTTAT
ARF18	ATCCAACAGTGCCTGAATTACG	AGAAGAAGAAGTAGAAGGAGAGGAA
BRL3	AAGTCACGAGAGGGCATA	CGTCTCACGCTTCCATAA
BZR1	CGTTACTAAGCGTAGTTATCCTCTG	CTGGAATCGAAGAAGAAGAATCTCT
DWF4	CGCTCAAAGTATGTTATCTAGTAGG	AGATTATTACCGAACTGGACAGTC
EDS1 (control)	TGGCTTTTCGTAGAAATTTCCC	GGAACCGGTTCGATTTCTCTC
