# Supplementary material for: Ca2+/Calmodulin-Dependent AtSR1/CAMTA3 Plays Critical Roles in Balancing Plant Growth and Immunity
Source: Int J Mol Sci. 2018 Jun 14;19(6):1764. doi: 10.3390/ijms19061764 (PMC6032152; doi:10.3390/ijms19061764)
Supplement: Supplementary file 1 [file ijms-19-01764-s001.zip › ijms-308896 supplimentory figure 1-4.pdf]

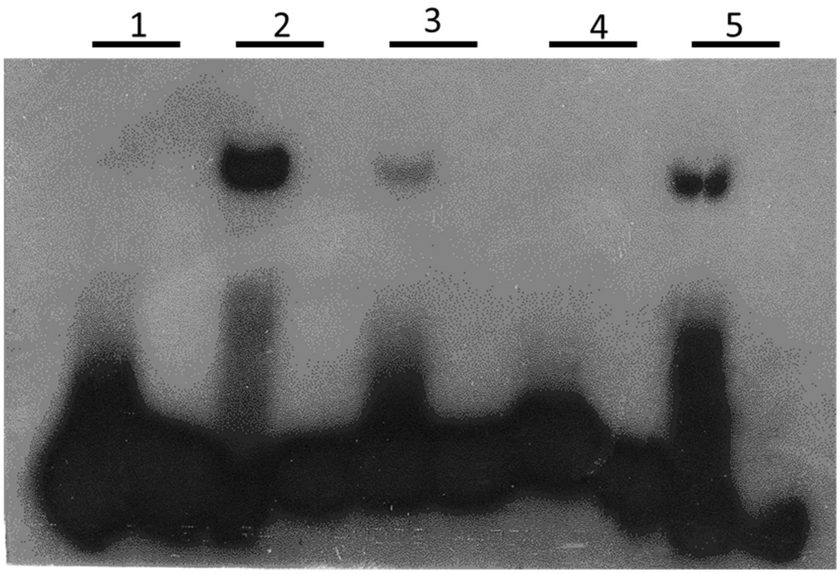

AtSR1 protein	+	-	+	-	+	-	+	-	+	-
DNA sequence	+	+	+	+	+	+	+	+	+	+

1. ADR1-L2 (+): ATTTTATTGAACGCGTGTTAAGGACG
2. At5g66890 (+): CTTCAAAGTACCGCGTGACTTTTCCG
3. At5g41750-3 (+): TTCTCATTTTACGCGTTTTTGCTTCA
4. At5g45000 (-): ATCTACCAGATCGCGGGAATGAGCAG
5. RLP32 (+): TTCCTCCAAACCGCGTTGGTTCTTCA

Figure S1. Promoter fragments of defense-related genes and purified recombinant AtSR1 protein were used in EMSA assay.

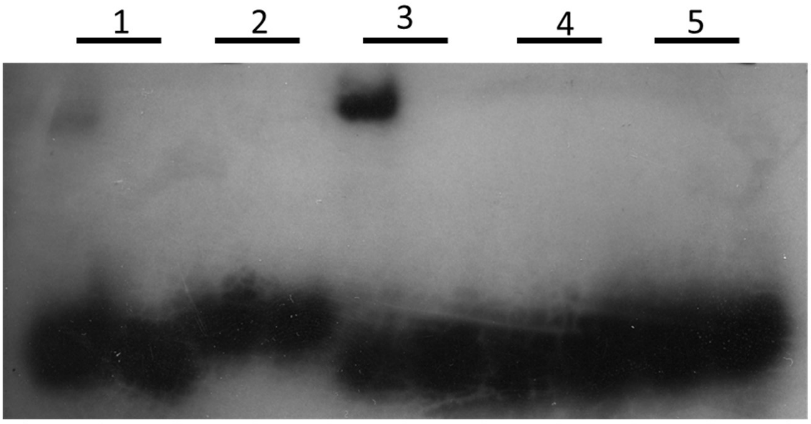

AtSR1 protein	+	-	+	-	+	-	+	-	+	-
DNA sequence	+	+	+	+	+	+	+	+	+	+

1. At1g56510 (+): ACGCTTCATTACGCGTTTTTGCATCC
2. NIMIN1-1 (-): ATAAATCGTCCCGCGATATACCGCGG
3. NIMIN1-2 (+): CCGCGATATACCGCGGGTTAAAATCT
4. NIMIN1-3 (-): ATAAATCGTCCCGCGATATACCGCGGGTTAAAATCT
5. NIMIN1-4 (-): GTTGATATCTCCGCGTTCTTTTACTC

Figure S2. Promoter fragments of defense-related genes and purified recombinant AtSR1 protein were used in EMSA assay.

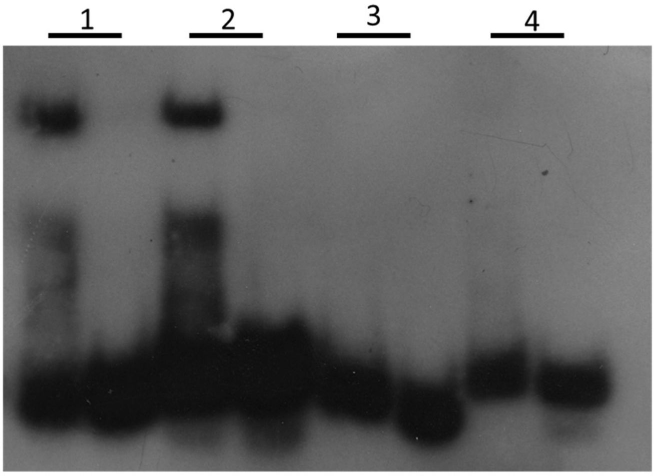

AtSR1 protein	+	-	+	-	+	-	+	-
DNA sequence	+	+	+	+	+	+	+	+

1. AFR18-1 (+): TCCCAAAACGCCGCGTGACCCTTCGT
2. AFR18-2 (+): CTAGTGTTTACCGCGTGAAGGTTTTT
3. IAA-1 (-): GGCTAGGATCTCGCGTACGCAACTGG
4. BZR1 (-): GTTTCCCAACTCGCGTTGATGGTTCG

Figure S3. Promoter fragments of growth-related genes and purified recombinant AtSR1 protein were used in EMSA assay.

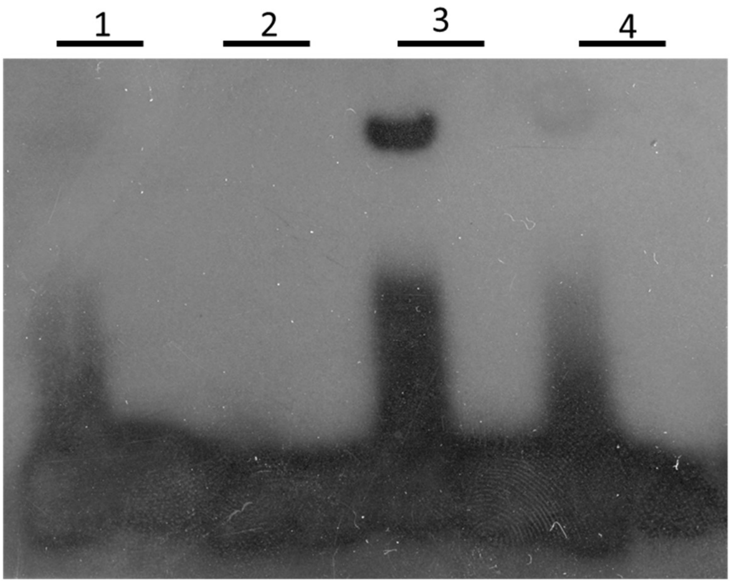

AtSR1 protein	+	-	+	-	+	-	+	-
DNA sequence	+	+	+	+	+	+	+	+

1. At5g35735-1 (+): ATATAATTGACCGCGTCAACATTTCA
2. At5g35735-2 (-): AAAAAAGCATCCGCGTTTGCTTTTCT
3. SAUR9 (+): TTACGTACTTACGCGGCTTTGATTTG
4. BRL3 (+): AGTTACATACACGCGTGGAAAAATAC

Figure S4. Promoter fragments of growth-related genes and purified recombinant AtSR1 protein were used in EMSA assay.
